# Association between FOXP3 promoter polymorphisms and cancer risk: A meta-analysis

**DOI:** 10.3892/ol.2014.2585

**Published:** 2014-10-02

**Authors:** LING-LING JIANG, LI-WEI RUAN

**Affiliations:** 1 Department of Neurology, Suzhou Municipal Hospital, Affiliated Suzhou Hospital of Nanjing Medical University, Suzhou, Jiangsu 215002, P.R. China; 2 Department of General Surgery (Breast and Thyroid Surgery), Shaoxing People’s Hospital, Shaoxing Hospital of Zhejiang University, Shaoxing, Zhejiang 312000, P.R. China

**Keywords:** FOXP3, polymorphism, cancer, meta-analysis

## Abstract

Epidemiological studies have been conducted to investigate the association between the FOXP3 promoter polymorphisms, rs3761549 and rs3761548, and the risk of cancer. However, the results from these studies have been controversial. In order to obtain a more precise conclusion of this association, the present meta-analysis was performed. The odds ratio (OR) and 95% confidence interval (95% CI) values were used to assess any correlations between the data. Overall, the rs3761549 (C>T) and rs3761548 (C>A) polymorphisms of the FOXP3 gene were not associated with the cancer risk in an Asian population. In the subgroup analyses based on cancer type, no significant associations were identified between these two polymorphisms and breast cancer. However, the results altered when the analyses were restricted to hepatocellular carcinoma (HCC) and non-small cell lung cancer (NSCLC) (for rs3761549: TT+CT vs. CC OR, 0.52, 95% CI, 0.38–0.72; TC vs. CC OR, 0.25, 95% CI, 0.16–0.39; T vs. C OR, 0.76, 95% CI, 0.59–0.97. For rs3761548: AA vs. AC+CC OR, 3.20, 95% CI 1.76–5.81; AA+AC vs. CC OR, 2.56, 95% CI, 1.75–3.76; AA vs. CC OR, 4.41, 95% CI, 2.36–8.25; AC vs. CC OR, 2.15, 95% CI, 1.42–3.25; A vs. C OR, 2.32, 95% CI, 1.74–3.10). The present meta-analysis indicates that the FOXP3 rs3761549 (C>T) and rs3761548 (C>A) polymorphisms are not associated with the risk of breast cancer, but with the risk of HCC and NSCLC. Therefore, a study with a larger sample size is required to further evaluate this association.

## Introduction

Cancer is a worldwide public health problem, which results from a complex interaction between environmental and genetic factors (1). Several polymorphic genes that are directly involved in tumorigenesis have also been proposed to contribute to the individual susceptibility to cancer (2).

The host immune defense has been shown to play a vital role in modulating human carcinogenesis (3). Regulatory T cells aid in keeping the balance between immunity and autotolerance, and are mainly characterized by CD4^+^/FOXP3^+^ or CD4^+^/CD25^+^/FOXP3^+^ expression. FOXP3 belongs to the forkhead family of transcription factors, and is involved in the regulation, activation and differentiation of T cells (4). In fact, the absence of a functional FOXP3 gene product has been revealed to cause an abnormal production of regulatory T cells (5). In addition, the loss of expression and somatic mutation of the human FOXP3 gene has been identified in human prostate and breast cancers. This suggests that FOXP3 may be a tumor suppressor and that inactivation of the FOXP3 gene may contribute to the development of cancer in humans (6,7).

The FOXP3 gene is positioned at the Xp11.23 locus on the X chromosome and encodes the FOXP3 protein, which is expressed in epithelial cells from various organs, such as the lungs and the thymus (8–11). The promoter polymorphisms in the FOXP3 gene are considered to affect FOXP3 production and activity. The FOXP3 gene rs3761549 (C>T) and rs3761548 (C>A) polymorphisms, located on the promoter region of the FOXP3 gene, are two of the most common single nucleotide polymorphisms. Previous studies have investigated the association between the FOXP3 rs3761549 and rs3761548 polymorphisms and the cancer risk, however, they have yielded conflicting results (12–16). Therefore, the present meta-analysis was performed to evaluate the role of these two polymorphisms and their association with the risk of cancer.

## Materials and methods

### Publication search and inclusion criteria

A comprehensive literature search, using the keywords ‘FOXP3’, ‘polymorphism’ and ‘tumor or cancer’, was performed using the PubMed, EMBASE and Chinese Wanfang databases (last search updated in February 10, 2014). Additional eligible studies were identified by manually searching the reference lists of reviews and original articles. In the event that data were published in more than one article, only studies with the largest sample size were selected for. The selection criteria to identify an eligible study were as follows: i) Investigation of the rs3761549 (C>T) and rs3761548 (C>A) polymorphisms of the FOXP3 gene and cancer risk; ii) the use of a case-control design, based on unrelated individuals; and iii) sufficient genotype distributions for cases and controls, so that an odds ratio (OR) with a 95% confidence interval (CI) could be assessed.

### Data extraction

The two authors independently reviewed and extracted the required data. Disagreements were resolved through discussion among the authors to achieve a consensus. The following information was recorded for each study: First author, year of publication, country, ethnicity, cancer type and number of genotypes (Table I).

### Statistical analysis

The OR corresponding to the 95% CI was used to assess the association between the FOXP3 polymorphisms and the risk of cancer. In addition to this comparison among all subjects, a stratified analysis by cancer type was also performed. The statistical heterogeneity among studies was assessed using I^2^ statistics and the Q-test (17). In the absence of any obvious heterogeneity, the fixed-effects model (the Mantel-Haenszel method) was applied to estimate the summary OR. Otherwise, the random-effects model (the DerSimonian and Laird method) was used (18,19). Sensitivity analysis was performed to identify the effect that the data from each study had on the pooled OR. Finally, any publication bias was evaluated using a funnel plot. All of the statistical tests were performed using RevMan 5.0 software (The Cochrane Collaboration, Oxford, UK).

## Results

**Figure 1:**
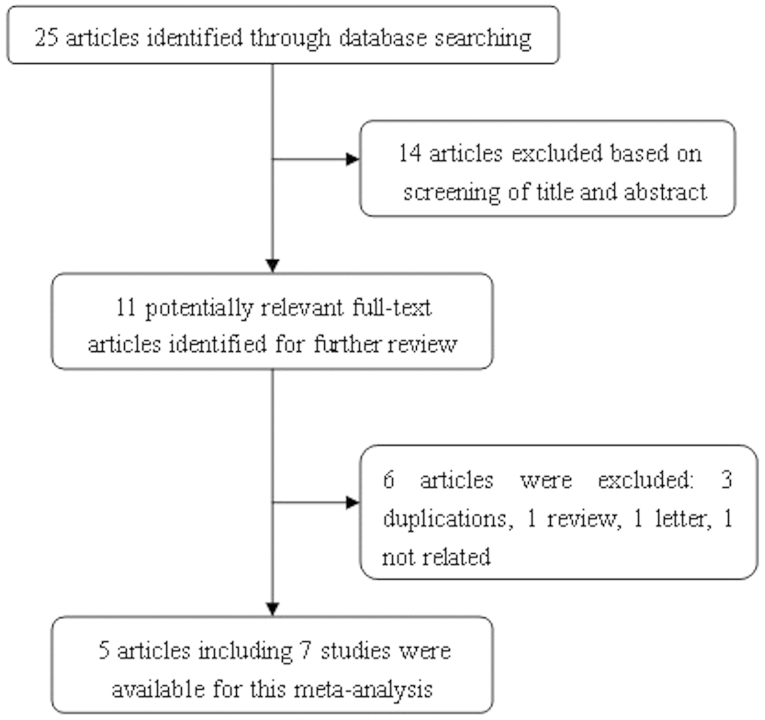
Process of identifying included studies.

**Figure 2:**
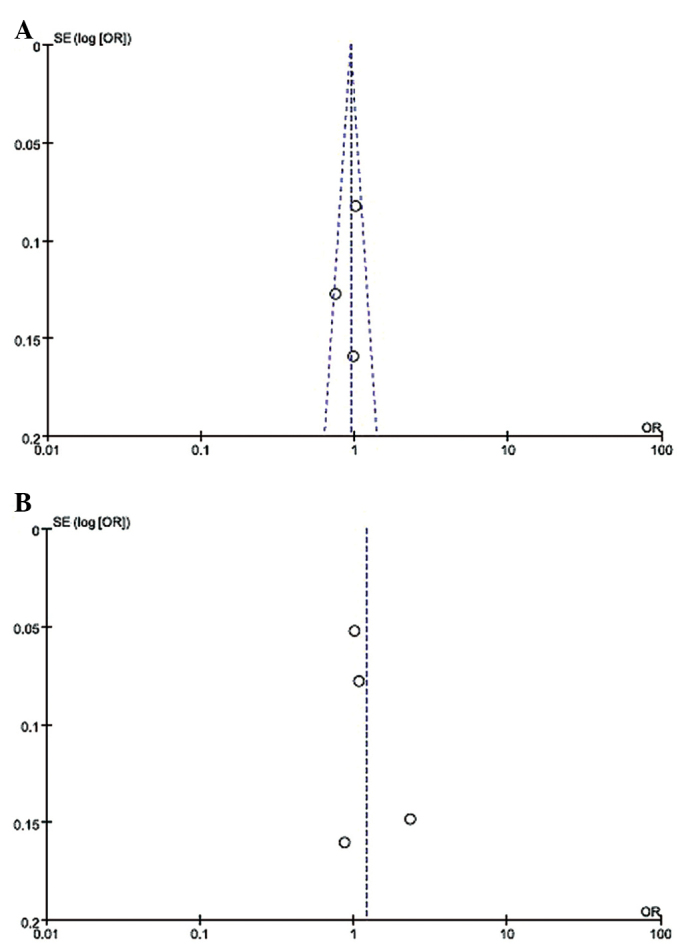
Funnel plot of the meta-analysis data to demonstrate the associations between the FOXP3 promoter polymorphisms and cancer risk. (A) rs3761549 T vs. C and (B) rs3761548 A vs. C. OR, odds ratio; SE, standard error.

The process of identifying suitable studies is shown in Fig. 1. A total of five studies (12–16), including 3,275 cases and 3,300 controls, were included in the present meta-analysis. All of the selected studies were based on Asian populations (Table I). The results of the pooled analysis revealed no significant association between the FOXP3 gene polymorphisms and the cancer risk (for rs3761549: TT vs. CT+CC OR, 1.20, 95% CI, 0.87–1.66; TT+CT vs. CC OR, 0.74, 95% CI, 0.41–1.33; TT vs. CC OR, 1.06, 95% CI, 0.76–1.46; TC vs. CC OR, 0.56, 95% CI, 0.17–1.80; T vs. C OR, 0.94, 95% CI, 0.83–1.06. For rs3761548: AA vs. AC+CC OR, 1.37, 95% CI, 0.87–2.16; AA+AC vs. CC OR, 1.18, 95% CI, 0.79–1.78; AA vs. CC OR, 1.36, 95% CI, 0.67–2.77; AC vs. CC OR, 1.11, 95% CI, 0.79–1.58; A vs. C OR, 1.21, 95% CI, 0.90–1.62). Further subgroup analysis was conducted based on cancer type, however, no association between the FOXP3 gene polymorphisms and the risk of breast cancer was revealed (for rs3761549: TT vs. CT+CC OR, 0.98, 95% CI, 0.60–1.60; TT+CT vs. CC OR, 1.01, 95% CI, 0.84–1.22; TT vs. CC OR, 0.98, 95% CI, 0.60–1.61; TC vs. CC OR, 1.02, 95% CI, 0.84–1.23; T vs. C OR, 1.01, 95% CI, 0.87–1.16. For rs3761548: AA vs. AC+CC OR, 1.09, 95% CI, 0.93–1.28; AA+AC vs. CC OR, 1.00, 95% CI, 0.88–1.12; AA vs. CC OR, 1.04, 95% CI, 0.86–1.26; AC vs. CC OR, 0.97, 95% CI, 0.86–1.10; A vs. C OR, 1.02, 95% CI, 0.94–1.11). However, statistical associations were observed with respect to hepatocellular carcinoma (HCC) and non-small cell lung cancer (NSCLC) (for rs3761549: TT+CT vs. CC OR, 0.52, 95% CI, 0.38–0.72; TC vs. CC OR, 0.25, 95% CI, 0.16–0.39; T vs. C: OR, 0.76, 95% CI, 0.59–0.97. For rs3761548: AA vs. AC+CC OR, 3.20, 95% CI, 1.76–5.81; AA+AC vs. CC OR, 2.56, 95% CI, 1.75–3.76; AA vs. CC OR, 4.41, 95% CI, 2.36–8.25; AC vs. CC OR, 2.15, 95% CI, 1.42–3.25; A vs. C OR, 2.32, 95% CI, 1.74–3.10) (Table II). The funnel plot, which assessed publication bias of the literature, appeared symmetrical in all of the genetic models (Fig. 2).

## Discussion

The characterization and identification of genes involved in the genetic predisposition and progression of cancer are critical for clinical practice and basic medical research. FOXP3 is an immunological regulator, and is able to repress oncogenes whilst activating additional tumor suppressor genes (6,20–22). FOXP3-mediated gene regulation follows the histone code of gene activation and suppression and alters histone modifications by binding to gene promoters (23,24). Epidemiological studies suggest that the FOXP3 promoter polymorphisms, rs3761549 and rs376154, are associated with the cancer risk. However, the results from these studies are conflicting. To provide a more detailed overview of the association, five genetic models were used in the current meta-analysis.

To the best of our knowledge, this was the first meta-analysis to provide comprehensive insight into the association between the FOXP3 polymorphisms and the risk of cancer. It was identified that the FOXP3 rs3761549 (C>T) and rs3761548 (C>A) polymorphisms were not associated with the risk of cancer among an Asian population. In addition, subgroup analysis revealed that the FOXP3 gene rs3761549 (C>T) and rs3761548 (C>A) polymorphisms were not associated with the risk of breast cancer. However, the rs3761549 (C>T) and rs3761548 (C>A) polymorphisms were linked with the risk of HCC and NSCLC, respectively. The results therefore indicated that the rs3761549 (C>T) and rs3761548 (C>A) polymorphisms may have a varying effect on carcinogenesis within different organs. However, these findings must be viewed with caution, since studies on HCC and NSCLC are rare. Therefore, the results from the present study may be due to chance.

There were certain limitations of this meta-analysis. Firstly, a relatively small number of studies and subjects were included, which could reduce the statistical power of the analysis. Secondly, the results were based on unadjusted estimates. A more precise analysis could be conducted if individual data were available. Thirdly, all published studies were based on Asian populations. Therefore, the results of this meta-analysis may be applicable to the specified ethnicity alone.

In conclusion, the present study demonstrated that the rs3761549 (C>T) and rs3761548 (C>A) polymorphisms in the promoter region of the FOXP3 gene were not associated with breast cancer, but instead were associated with HCC and NSCLC. Therefore, a future study that consists of a larger sample size is required to further evaluate this association.

## Figures and Tables

**Table I:** Characteristics of studies included in the present meta-analysis.

					rs3761549		rs3761548	
						
First author (ref.)	Year	Country	Ethnicity	Cancer type	Case (TT/CT/CC)	Control (TT/CT/CC)	Case (AA/AC/CC)	Control (AA/AC/CC)
Chen *et al* (12)	2013	China	Asian	Hepatocellular carcinoma	59/28/301	41/88/233	-	-
He *et al* (13)	2013	China	Asian	Non-small cell lung cancer	-	-	37/80/75	18/80/161
Jahan *et al* (14)	2013	India	Asian	Breast cancer	0/198/4	0/128/2	27/160/15	20/106/4
Raskin *et al* (15)	2009	Israel	Asian	Breast cancer	-	-	320/722/402	303/763/392
Zheng *et al* (16)	2013	China	Asian	Breast cancer	32/283/734	34/290/767	38/338/673	30/342/719

**Table II:** Meta-analysis data of the associations between the FOXP3 promoter polymorphisms and the cancer risk in all genetic models.

A, rs3761549 polymorphism										

	TT vs. CT+CC		TT+CT vs. CC		TT vs. CC		TC vs. CC		T vs. C	
					
Variables	OR (95% CI)	P-value	OR (95% CI)	P-value	OR (95% CI)	P-value	OR (95% CI)	P-value	OR (95% CI)	P-value
Total	1.20 (0.87–1.66)	0.28	0.74 (0.41–1.33)	0.01	1.06 (0.76–1.46)	0.71	0.56 (0.17–1.80)	0.01	0.94 (0.83–1.06)	0.15
Breast cancer	0.98 (0.60–1.60)	-	1.01 (0.84–1.22)	0.76	0.98 (0.60–1.61)	-	1.02 (0.84–1.23)	0.75	1.01 (0.87–1.16)	0.92
HCC	1.40 (0.92–2.15)	-	**0.52 (0.38–0.72)**	-	1.11 (0.72–1.72)	-	**0.25 (0.16–0.39)**	**-**	**0.76 (0.59–0.97)**	**-**

B, rs3761548 polymorphism										

	AA vs. AC+CC		AA+AC vs. CC		AA vs. CC		AC vs. CC		A vs. C	
					
Variables	OR (95% CI)	P-value	OR (95% CI)	P-value	OR (95% CI)	P-value	OR (95% CI)	P-value	OR (95% CI)	P-value

Total	1.37 (0.87–2.16)	0.01	1.18 (0.79–1.78)	0.01	1.36 (0.67–2.77)	0.01	1.11 (0.79–1.58)	0.01	1.21 (0.90–1.62)	0.01
Breast cancer	1.09 (0.93–1.28)	0.53	1.00 (0.88–1.12)	0.16	1.04 (0.86–1.26)	0.14	0.97 (0.86–1.10)	0.18	1.02 (0.94–1.11)	0.45
NSCLC	**3.20 (1.76–5.81)**	**-**	**2.56 (1.75–3.76)**	**-**	**4.41 (2.36–8.25)**	**-**	**2.15 (1.42–3.25)**	**-**	**2.32 (1.74–3.10)**	**-**

Bold indicates significant results for the association between FOXP3 promoter polymorphisms and cancer risk. HCC, hepatocellular carcinoma; NSCLC, non-small cell lung cancer; OR, odds ratio; 95% CI, 95% confidence interval; P-value, P-value for heterogeneity.
